# Multiplexed, High-Sensitivity Measurements of Antibody Affinity Using Interferometric Reflectance Imaging Sensor

**DOI:** 10.3390/bios11120483

**Published:** 2021-11-27

**Authors:** Allison M. Marn, James Needham, Elisa Chiodi, M. Selim Ünlü

**Affiliations:** 1 School of Engineering, Computing, and Construction Management, Roger Williams University, Bristol, RI 02809, USA; 2 InBios International, Inc., Seattle, WA 98109, USA; james@inbios.com; 3 Department of Electrical Engineering, Boston University, Boston, MA 02215, USA; elich@bu.edu (E.C.); selim@bu.edu (M.S.Ü.); 4 Department of Biomedical Engineering, Boston University, Boston, MA 02215, USA

**Keywords:** multiplexed antibody kinetics, IRIS, interferometric sensing, anthrax lethal factor

## Abstract

Anthrax lethal factor (LF) is one of the enzymatic components of the anthrax toxin responsible for the pathogenic responses of the anthrax disease. The ability to screen multiplexed ligands against LF and subsequently estimate the effective kinetic rates ($k_{on}$ and $k_{off}$) and complementary binding behavior provides critical information useful in diagnostic and therapeutic development for anthrax. Tools such as biolayer interferometry (BLI) and surface plasmon resonance imaging (SPRi) have been developed for this purpose; however, these tools suffer from limitations such as signal jumps when the solution in the chamber is switched or low sensitivity. Here, we present multiplexed antibody affinity measurements obtained by the interferometric reflectance imaging sensor (IRIS), a highly sensitive, label-free optical biosensor, whose stability, simplicity, and imaging modality overcomes many of the limitations of other multiplexed methods. We compare the multiplexed binding results obtained with the IRIS system using two ligands targeting the anthrax lethal factor (LF) against previously published results obtained with more traditional surface plasmon resonance (SPR), which showed consistent results, as well as kinetic information previously unattainable with SPR. Additional exemplary data demonstrating multiplexed binding and the corresponding complementary binding to sequentially injected ligands provides an additional layer of information immediately useful to the researcher.

## 1. Introduction

Anthrax lethal toxin (LTx) is a toxin produced by bacillus anthracis, a bacterium commonly found naturally in soil [1]. This toxic compound can persist in the bloodstream, even after antibiotic treatment, leading to severe disease and sometimes death [1]. Anthrax lethal factor (LF) is one of the two components comprising LTx [2]. Developing antibody-based assays for LF is crucial for determining the most specific antibodies to target this compound, the first step towards the development of new treatments for the neutralization of the toxin [3].

Here we have utilized the interferometric reflectance imaging sensor (IRIS) to characterize anti-LF ligands and compare the results with surface plasmon resonance (SPR). While SPR is currently the gold standard in binding kinetic measurements, the screening potential of SPR is limited by the number of channels in the device and, therefore, lacks the ability to perform highly multiplexed measurements for screening multiple ligands simultaneously. Surface plasmon resonance imaging (SPRi) and biolayer interferometry (BLI) provide useful tools for high throughput measurements of binding kinetics; however, there are some limitations to their use. SPRi provides multiplexed binding information in a microarray based format, but the imaging SPR modality suffers from a higher noise floor, and therefore, lower sensitivity, than traditional SPR [4,5,6,7]. In addition, the functionalized gold surfaces that are required for SPRi and the requisite instrumentation are costly expenses, which, in addition to the time-consuming analysis, may be a hindrance to researchers [8].

BLI provides a high throughput well-based method for screening multiple ligands; however, the need to typically functionalize reagents (e.g., biotinylate antibodies for BLI) [9] or use an intermediary antibody to sequester ligands to the probe surface may unduly influence the estimated binding kinetics. Furthermore, the absence of flow can introduce mass transport limitations, which may also undesirably alter the measured binding kinetics [10]. Additional limitations, including reagent mixing, sample evaporation, and physically moving the tip from one reagent well to another, may limit experimental protocols, complicate measurements, and impact the subsequent analysis for BLI methods [10,11].

The IRIS system uses the interference signal from a dual-layer substrate of SiO_2_/Si to measure changes in optical path difference due to biomass accumulation on the sensor surface. The principles describing this modality have been extensively discussed in Özkumur et al. [12] and Daaboul et al. [13]. Through this measurement of biomass accumulation, the IRIS system allows monitoring real-time binding events in an inexpensive array-based format [14]. The sensitivity of IRIS is comparable or exceeding that of competing technologies. BLI has a reported sensitivity of 10 pg/mm^2^ [15], and SPR instruments have a sensitivity in the range of 10–0.01 pg/mm^2^ [16]. We have recently reported a sensitivity with IRIS of 1 pg/mm^2^ in Chiodi et al. [17], which has since been improved to 0.2 pg/mm^2^ [18] using the methods described [17]. While the sensitivity of the detection method is crucial when selecting a biosensing technology for a particular application, the overall practical benefit of the method is also critical [19]. IRIS combines comparable sensitivity to SPR with higher multiplexing capability.

Multiplexed affinity measurements allow for the simultaneous analysis of multiple ligands targeting a variety of epitopes under the same experimental conditions. Performing a single multiplexed experiment saves on both time and the cost of reagents, as well as eliminating the potential for subtle variations between experiments impacting the comparative results. The effective kinetic rates ($k_{on}$ and $k_{off}$) and complementary binding behavior obtained from these multiplexed measurements provide critical information useful in diagnostic and therapeutic development.

Furthermore, the high sensitivity of IRIS allows for the use of a thin, quasi-2D functionalization layer for ligand immobilization. In contrast, SPR often uses a 3D hydrogel matrix to immobilize the ligand for increased sensitivity. While this allows for the immobilization of more ligands in the sensing volume, the analyte must diffuse through the hydrogel to interact with the ligand, which can limit the measured kinetics [20,21]. Additionally, IRIS, unlike SPR, does not require a reference channel to obtain accurate measurements, and, unlike BLI, there is no artificial jump in the acquired data as new buffers of similar refractive indices are injected over the chip surface [16,22].

Here, we compare the multiplexed binding of the anthrax lethal factor (LF) antigen to a panel of ligands and compare the results to previously measured binding rates determined by SPR with no multiplexing [23] using a straightforward 1:1 Langmuir binding model [24]. We further examined bivalent fitting for the determination of more accurate affinity constants as a demonstration of potential for improvements in kinetic analysis. Moreover, we analyze the signal-to-noise ratios of these binding signals to quantify the data quality. We then evaluate a larger panel of anti-LF ligands targeting first LTx and then LF and then monitor the complementary ligand binding to find potential binding pairs for both the whole toxin and its lethal factor.

## 2. Materials and Methods

### 2.1. IRIS Instrument

**Figure 1:**
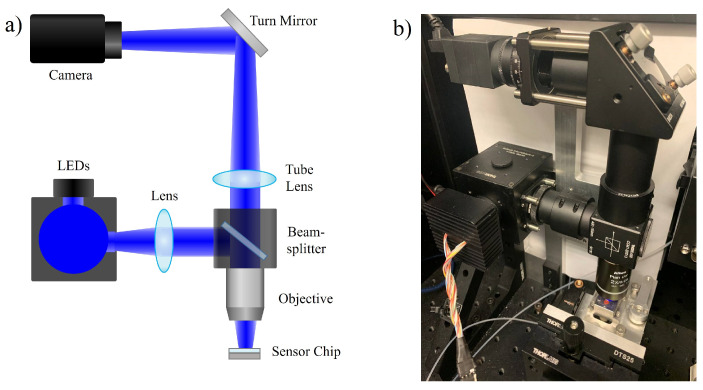
(**a**) A simplified diagram of the setup and (**b**) the actual physical IRIS instrument.

The physical setup of the IRIS instrument is schematically shown in Figure 1. The LED light source is sent into an integrating sphere (Thorlabs, IS236A-4). The exiting illumination is collected with an achromatic doublet lens towards a 50:50 beam splitter. Half of the light is reflected and focused on the IRIS chip assembly using a 2× objective. The reflected interferometric intensities are then collected by the objective and focused onto the camera sensor (FLIR, Grasshopper3 GS3-U3-51S5M-C) using a tube lens. A peristaltic pump is used to inject the samples into the chip assembly. While multiple LED wavelengths may be used for generating quantitative estimates of the accumulated mass (nm or pg/mm^2^), a single LED wavelength (central wavelength 456 nm) can be used where shifts in the reflected intensity are approximately directly proportional to the thickness change observed with the current IRIS chip.

### 2.2. IRIS Chip

The IRIS chip is composed of a Si-SiO_2_ substrate, functionalized with epoxysilane (3-(glycidoxipropyl) trimethoxysilane, GPTS) and spotted with a series of relevant antigens or antibodies using a benchtop microarray spotter (BioRad BioOdyssey Calligrapher™, Hercules, CA, USA) [25]. After spotting, the chips were incubated overnight before blocking with a 1% (*m*/*v*) bovine serum albumin (BSA) in a phosphate buffered saline solution, containing 0.1% tween-20 (1% BSA in PBST). An AR-coated glass slide with a pressure sensitive adhesive (PSA) gasket is attached to the top of the chip, and buffer enters and exits the assembly via two holes that have been laser etched through the chip surface.

### 2.3. Reagents

The purified recombinant LF antigen was provided by InBios International, Inc. (Seattle, WA, USA). Anthrax protective antigen, PA63, was purchased from List Biological Laboratories, Inc. (Campbell, CA, USA). Single-chain camelid antibodies (VHH) were developed at the Shoemaker Laboratory and screened for high affinity binding via ELISA and SPR [23] and subsequently tested at InBios International, Inc., using the IRIS system. LTx was formed by mixing LF and PA63 in a 5:1 mass ratio (15 μg/mL:3 μg/mL) in 1% BSA/PBST. All buffers were prepared using highly purified deionized water (>18 MΩ).

### 2.4. Assay Procedure

A chip was prepared to estimate the affinity constants for lethal factor (LF) binding to single-chain VHH by spotting relevant VHH ligands and controls using a benchtop microarray spotter (∼300 μm diameter spot sizes). After overnight incubation at room temperature, the chip was blocked and washed for 5 min with a buffer solution of 1% BSA/PBS and 0.1% tween-20 and gently dried with nitrogen gas. A spacer coated in pressure-sensitive adhesive and cover glass were pressed onto the chip to form a microfluidic cartridge, which was loaded into the IRIS reader. A solution of 0.1 M glycine (pH 2.5) was injected over the chip surface to remove any loosely bound ligands before subsequently equilibrating the chip by injecting 1% BSA/PBST. A baseline signal for each spot was determined by flowing 1% BSA/PBST over the chip for a period of 5 min, while images were sequentially collected every ∼9 s. A solution of 10 μg/mL of LF antigen (diluted into 1% BSA/PBST) was then injected for 10 min to monitor the binding characteristics for each ligand. Dissociation kinetics were observed by then injecting the buffer only (1% BSA/PBST) over the chip surface for another 45 min.

Screening for lethal toxin (LTx) binding, lethal factor (LF) binding, and subsequent complementary ligand binding was performed in a similar manner. A large panel of anti-LF ligands and controls were spotted onto the chip surface using ∼100 μm diameter spot sizes. After the chip was spotted, blocked and assembled, buffer was injected to measure and record a baseline. LTx was then injected for 20 min, followed by 1% BSA/PBST for 30 min to monitor dissociation. Four additional ligands were then sequentially injected at 10 μg/mL concentrations for 10 min to monitor for complementary binding activity. The formed complexes were then eluted by injecting 0.1 M glycine, pH 2.5, before equilibrating with 1% BSA/PBST. LF antigen (10 μg/mL) was then injected for 20 min, followed by only buffer for 30 min. Two additional anti-LF solutions were injected sequentially to monitor for complementary binding activity.

### 2.5. Analysis

Image slices were collected (using ImageJ software) every ∼9 s, representing an average of 90 individual images. Ninety frames are averaged to reduce noise in the imaging system; the effect of image averaging in minimizing noise in the IRIS system has been discussed further in [17]. As binding occurs, the reflected intensity measured increases where biomass has accumulated. To visualize binding, the first image slice can be used as a reference image (Iref), subtracting all subsequent images from this reference image time point to create a “difference” image.

Binding curves are generated by recording the average intensity within the spot and subtracting the average reflected intensity in a neighboring area just outside of the spot of interest. Estimates for $k_{on}$, $k_{off}$, and $K_{D}$ were determined by globally fitting the raw data to a simple 1:1 Langmuir binding curve using a custom Python script with an interactive UI. More sophisticated analyses may be applicable with these data sets (e.g., bivalent fits, surface inhomogeneity fits, etc.; see [20]), but as the reference SPR data was fit with a 1:1 model, we report results with a similar approach in order to verify assay results.

## 3. Results

### 3.1. Estimates of Affinity Constants for Anti-LF Ligands

**Figure 2:**
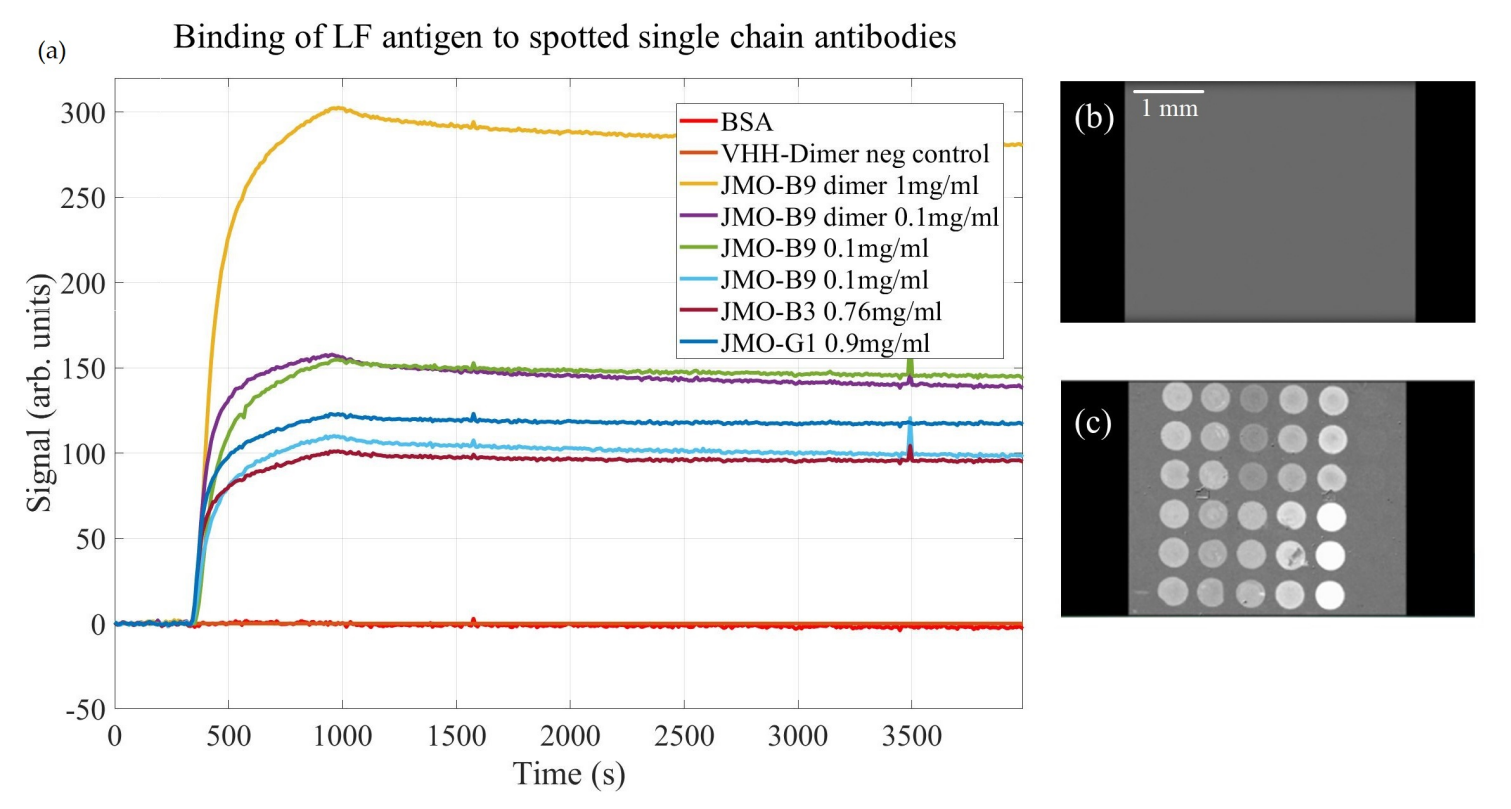
The raw binding curve data for VHH ligands targeting LF is shown above. In (**a**), the LF antigen (10 μg/mL) was injected at the indicated time point and buffer was subsequently injected at the indicated time point to monitor dissociation. Images (**b**,**c**) show the “difference images” at the starting point (no visible binding) and during the dissociation period (significant specific binding observed with anti-LF VHH ligands).

The raw data for VHH ligand binding to injected LF antigen is shown in Figure 2a. Example “difference” image slices are shown Figure 2b, the initial image, and Figure 2c, the final image. The image of the spotted chip with reagent locations labeled is included as Figure S1 in the Supplementary Material. Raw binding curves for the single-chain antibodies were normalized to the VHH-dimer negative control before performing any kinetic analysis.

The dynamic binding curves for JMO-B3, JMO-B9, and JMO-G1 were fit to a 1:1 model to estimate $k_{on}$, $k_{off}$, and $K_{D}$. These estimates are shown in Table 1 alongside the previously published parameters generated with SPR [23]. Of note, the JMO-B9 had no reported SPR estimates as binding was not observed via SPR despite binding being observed on ELISA, which may have been due to steric hindrance as the LF antigen [23]. The LF antigen is covalently attached through amine coupling chemistry to the functionalization layer for SPR and may not have presented an appropriate epitope for the JMO-B9. We note that both the measured $k_{on}$ and $k_{off}$ values were lower than those obtained via SPR, but the overall $K_{D}$ values were within the error bounds of the measurements.

**Figure 3:**
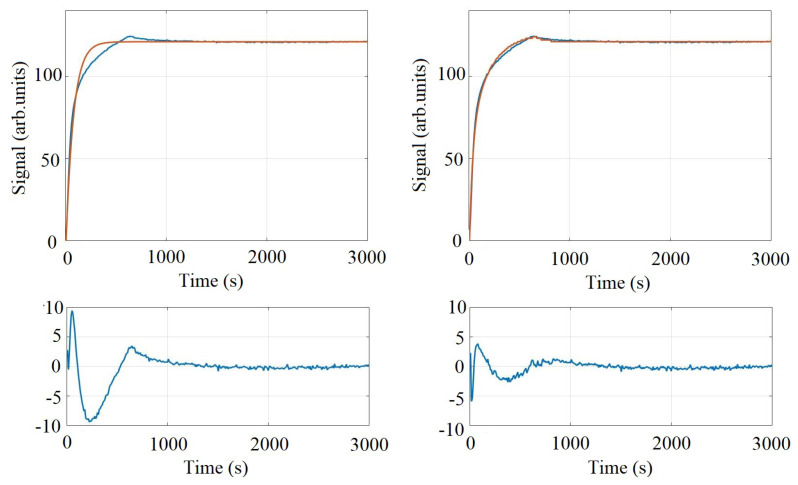
Fit curves for the average JMO-G1 signal are shown above for both a 1:1 Langmuir binding curve and a bivalent fit curve. Non-random error is apparent with the 1:1 fit, which is significantly reduced upon incorporating a bivalent fit model.

**Figure 4:**
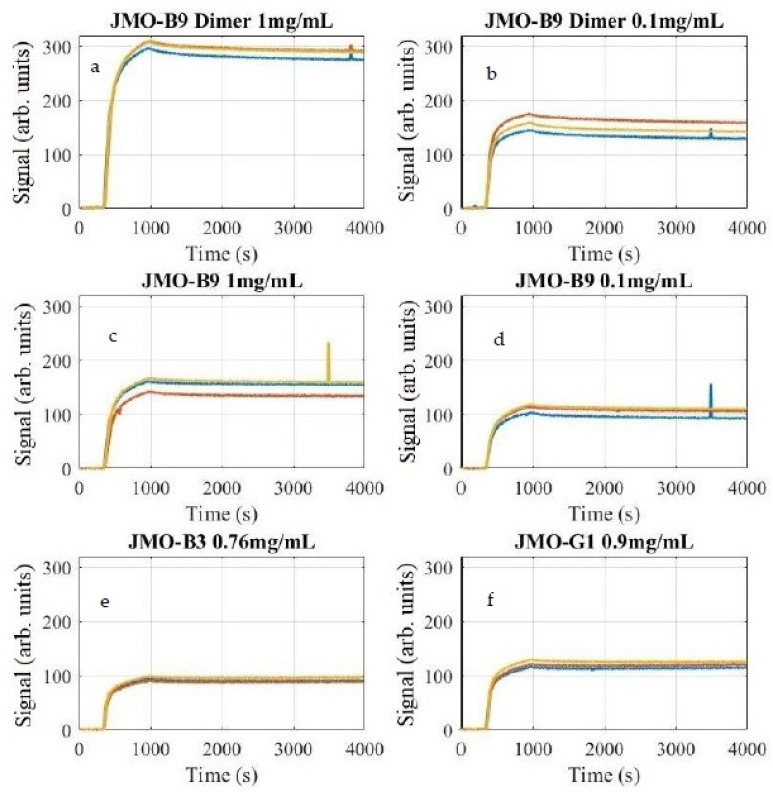
The individual signal from each spot for each reagent: (**a**) JMO-B9 Dimer 1 mg/mL, (**b**) JMO-B9 Dimer 0.1 mg/mL, (**c**) JMO-B9 1 mg/mL, (**d**) JMO-B9 0.1 mg/mL, (**e**) JMO-B3 0.76 mg/mL, and (**f**) JMO-G1 0.9 mg/mL.

The binding curves exhibit low noise characteristics, and the signal-to-noise ratio (SNR) and variability between spots can be seen in Table 2, with high SNR values ranging from 281 to 824. Additionally, an exemplary 1:1 fit model and a bivalent fit model are shown for the average JMO-G1 ligand in Figure 3. As can be seen, the non-random error for the 1:1 fit may indicate that the selected model may not be completely appropriate for the physics occurring at the sensor surface. The bivalent fit model significantly reduces the observed error, but the additional parameters available may also contribute to overfitting [20]. The signals corresponding to each spot of the microarray are shown in Figure 4 grouped by reagent. Artifacts present in the signal that appear as a small spike were determined by the images to be due by a small bubble in the fluidic system.

### 3.2. Multiplexed LTx and LF Binding

**Figure 5:**
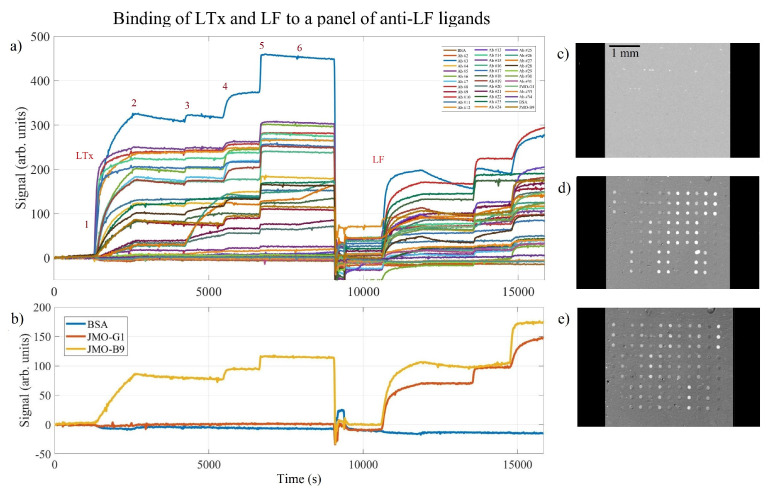
(**a**) The average binding signal for each of the spotted anti-LF ligands. Potential binding pairs were evaluated after injecting lethal toxin (LTx) or lethal factor (LF) and then serially injecting anti-LF antibodies over the chip surface. The first section begins with the injection of LTx, numbers 1–6 indicate, respectively, the injection of LTx, running buffer only, injecting complementary Ab#1, complementary Ab#2, complementary Ab#3, and complementary Ab#4. A similar process is then followed during the LF portion of the assay. (**b**) Selected ligands (binding curves) from (**a**) are shown separately for clarity, illuminating that JMO-B9 binds to both LTx and LF antigens, while JMO-G1 appears to bind to only the purified recombinant LF antigen. The individual “difference” image slices are shown, indicating the starting point (**c**) and the binding observed after injecting LTx (**d**) and after injecting LF (**e**) for several ligands.

A larger panel of anti-LF ligands were spotted onto a single IRIS chip. After overnight incubation at room temperature, the chip was blocked and washed for 5 min with a buffer solution of 1% BSA/PBS and 0.1% tween-20 and gently dried with nitrogen gas. The microfluidic chamber was formed by pressing a spacer coated in pressure-sensitive adhesive and a cover glass onto the chip. The cartridge was then loaded into the IRIS reader for the injection of the analytes. Spot sizes were ∼100 μm in diameter, and all proteins were spotted in triplicate. The image of the spotted chip with reagent locations labeled is included as Figure S2 in the Supplementary Material. Raw binding curve data for the panel of anti-LF ligands are shown in Figure 5a and represent the average of the triplicate spots. A variety of binding rates are evident here, and the sequential addition of anti-LF antibodies demonstrated potential binding pairs for LTx first and then LF.

As can be seen in Figure 5a, individual “steps” are observed as potentially complementary antibodies are injected over the chip surface. This may aid in the selection of appropriate binding pairs during immunoassay development. Careful observation of the binding curves in Figure 5a and “difference” images seen in Figure 5b–d indicate some ligands successfully bound to LF but not the complex formation of LTx [26], perhaps indicative that an epitope present on LF may be hidden upon the formation of the toxin.

The selected reagents, JMO-B9 and JMO-G1, are graphed separately in Figure 5b, where markedly different binding behavior can be seen for each reagent. The JMO-B9 binds to both the LTx complex and the LF antigen, whereas the JMO-G1 appears to bind solely to LF and not to the LTx complex. Since LTx is comprised of a protective antigen (PA) and LF and binding is observed to the isolated LF antigen but not LTx, this suggests that the epitope where JMO-G1 binds is likely blocked by the presence of the PA in the LTx complex, demonstrating the utility of this screening method for providing insight into the preferential binding of molecules.

## 4. Discussion

The IRIS has demonstrated significant capability in monitoring real-time binding events to estimate the kinetic rates for antibody–antigen interactions. Measurements of the $k_{on}$ and $k_{off}$ values from the dynamic binding of the LF antigen to two different single-chain antibodies were reported with favorable comparisons to results obtained via SPR. Given significant differences in experimental conditions, such as the LF antigen being attached to the surface for SPR measurements, a significant correlation of the kinetic rates was observed. We do note that both the $k_{on}$ and $k_{off}$ rates were somewhat reduced relative to the SPR measurements, which may be indicative of mass transport limitations. Additionally, one anti-LF VHH ligand (JMO-B9) that was unable to be measured via traditional SPR produced meaningful binding results with IRIS.

Multiplexed binding events were also demonstrated with a large panel of anti-LF ligands. Binding to both LTx and LF were shown with additional complementary antibodies injected at sequential time points in order to evaluate potential binding pairs. Significant differences in the observed binding behavior for select ligands was indicative of preferential binding for LF over LTx.

The utility, simplicity of operation, and inexpensive methodology of IRIS, combined with its ability to simultaneously screen multiple ligands for binding kinetics and potential binding pairs as shown here, prove IRIS to be a useful and accessible method for the selection and optimization of ligand-analyte interaction.

## Figures and Tables

**Table 1:** The fit kinetic parameters with the IRIS system are shown side-by-side with the previously published SPR results. JMO-B9 had no data reported using the SPR system. Error bounds for the IRIS fit kinetic parameters represent ±1 standard deviation of the values obtained from curve fitting each of the replicate spots for each ligand.

	Published Values 1:1 Fit			IRIS Kinetic Parameters 1:1 Fit		
	(Vrentas et al., 2016)					
Reagent	$k_{\mathrm{on}}$ $\left(M^{-1}s^{-1}\right)$	$k_{\mathrm{off}}$ $\left(s^{-1}\right)$	$K_{D}$ (M)	$k_{\mathrm{on}}$ $\left(M^{-1}s^{-1}\right)$	$k_{\mathrm{off}}$ $\left(s^{-1}\right)$	$K_{D}$ (M)
JMO-B3	7.7±1.5 $\times 10^{5}$	7.6±0.5 $\times 10^{-6}$	2.0±0.5 $\times 10^{-11}$	1.4±0.13 $\times 10^{5}$	5.0±2.5 $\times 10^{-6}$	3.6±1.5 $\times 10^{-11}$
JMO-B9	N/A	N/A	N/A	7.5±0.62 $\times 10^{4}$	1.5±0.32 $\times 10^{-5}$	2.0±0.6 $\times 10^{-10}$
JMO-G1	5.5±0.6 $\times 10^{5}$	6.6±3.0 $\times 10^{-6}$	1.1±0.5 $\times 10^{-11}$	1.33±0.17 $\times 10^{5}$	4.4±1.9 $\times 10^{-6}$	3.3±1.8 $\times 10^{-11}$

**Table 2:** The signal-to-noise ratio measured as the binding signal over the standard deviation of the average signal of the three spots for each reagent and the average standard deviation of each spot from the average signal normalized by the binding signal are shown here.

Reagent	Signal/Noise	Mean Standard Deviation/Signal
JMO-B9 Dimer 1 mg/mL	824	0.028
JMO-B9 Dimer 0.1 mg/mL	262	0.095
JMO-B9 1 mg/mL	381	0.089
JMO-B9 0.1 mg/mL	281	0.077
JMO-B3 0.76 mg/mL	479	0.096
JMO-G1 0.9 mg/mL	433	0.056

## Supplementary Materials

The following are available at https://www.mdpi.com/article/10.3390/bios11120483/s1. Figure S1. IRIS image showing the spotting scheme used for the experiments show in Figure 2, Figure 3 and Figure 4. Figure S2. IRIS image showing the spotting scheme used for the experiments show in Figure 5.
