# Supplementary material for: Multiplexed, High-Sensitivity Measurements of Antibody Affinity Using Interferometric Reflectance Imaging Sensor
Source: Biosensors (Basel). 2021 Nov 27;11(12):483. doi: 10.3390/bios11120483 (PMC8699213; doi:10.3390/bios11120483)
Supplement: Supplementary file 1 [file biosensors-11-00483-s001.zip › biosensors-1413479-supplementary.pdf]

biosensors

Supporting Information

Multiplexed, High-Sensitivity Measurements of Antibody Affinity Using Interferometric Reflectance Imaging Sensor

Allison M. Marn [1,*], James Needham [2], Elisa Chiodi [3] and M. Selim Ünlü [3,4]

[1] School of Engineering, Computing, and Construction Management, Roger Williams University, Bristol, RI 02809, USA
[2] InBios International, Inc., Seattle, WA 98109, USA; james@inbios.com
[3] Department of Electrical Engineering, Boston University, Boston, MA 02215, USA; elich@bu.edu (E.C.); selim@bu.edu (M.S.Ü.)
[4] Department of Biomedical Engineering, Boston University, Boston, MA 02215, USA
* Correspondence: amarn@rwu.edu

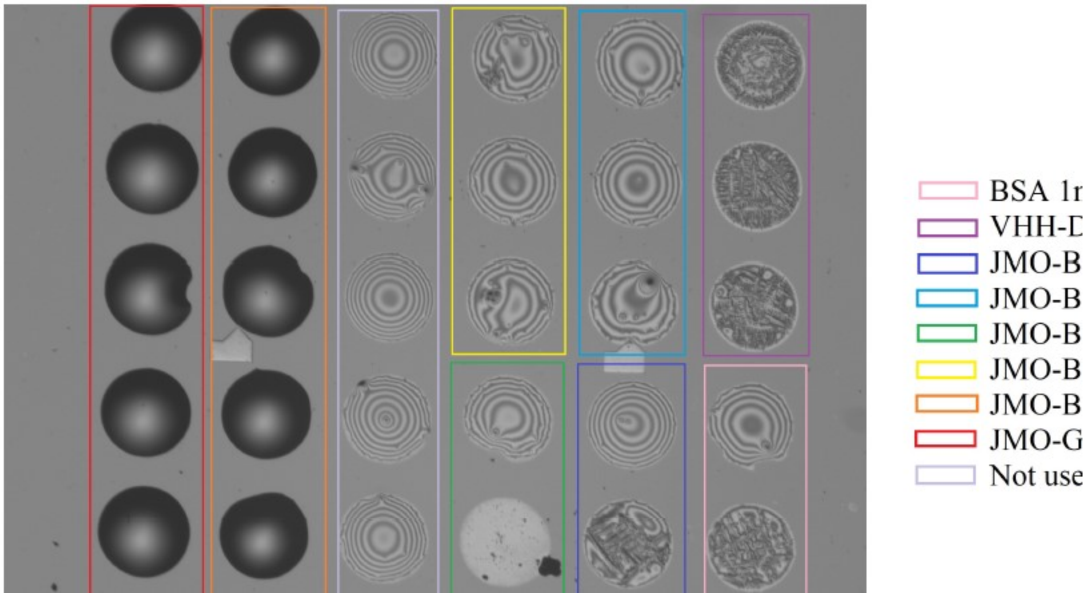

Figure S1. IRIS image showing the spotting scheme used for the experiments show in Figures 2–4.

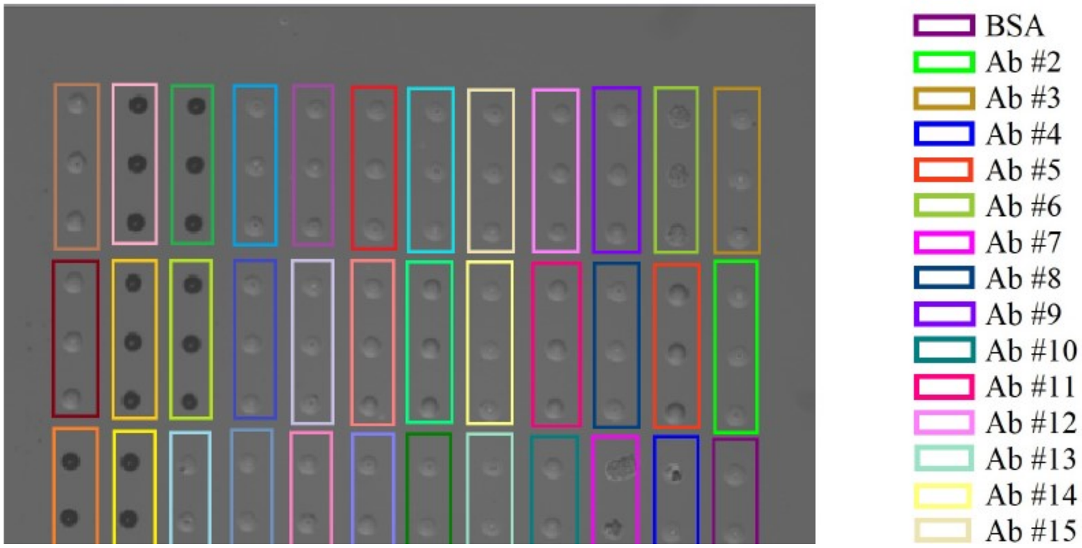

Figure S2. IRIS image showing the spotting scheme used for the experiments show in Figure 5.
